# Effects of medicaid expansion on poverty disparities in health insurance coverage

**DOI:** 10.1186/s12939-021-01486-3

**Published:** 2021-07-26

**Authors:** Yilu Lin, Alisha Monnette, Lizheng Shi

**Affiliations:** Department of Health Policy and Management, School of Public Health and Tropical Medicine, Tulane University, 1440 Canal Street, Suite 1900, Louisiana 70112 New Orleans, USA

**Keywords:** Health equity, Medicaid expansion, Poverty disparity, Insurance coverage

## Abstract

**Background:**

More than 30 states have either expanded Medicaid or are actively considering expansion. The coverage gains from this policy are well documented, however, the impacts of its increasing coverage on poverty disparity are unclear at the national level.

**Method:**

American Community Survey (2012–2018) was used to examine the effects of Medicaid expansion on poverty disparity in insurance coverage for nonelderly adults in the United States. Differences-in-differences-in-differences design was used to analyze trends in uninsured rates by poverty levels: (1) < 138 %, (2) 138–400 % and (3) > 400 % federal poverty level (FPL).

**Results:**

Compared with uninsured rates in 2012, uninsured rates in 2018 decreased by 10.75 %, 6.42 %, and 1.11 % for < 138 %, 138–400 %, and > 400 % FPL, respectively. From 2012 to 2018, > 400 % FPL group continuously had the lowest uninsured rate and < 138 % FPL group had the highest uninsured rate. Compared with ≥ 138 % FPL groups, there was a 2.54 % reduction in uninsured risk after Medicaid expansion among < 138 % FPL group in Medicaid expansion states versus control states. After eliminating the impact of the ACA market exchange premium subsidy, 3.18 % decrease was estimated.

**Conclusion:**

Poverty disparity in uninsured rates improved with Medicaid expansion. However, < 138 % FPL population are still at a higher risk for being uninsured.

## Background

Large disparities in health insurance coverage, related to poverty, have been a long-standing issue in the United States (US) and a significant concern among policymakers and health care professionals. According to the Kaiser Family Foundation, as of 2019, individuals under 200 % FPL accounted for 30 % of the total US population [1]. This large proportion of low-income individuals signifies a need to investigate the disparities in poverty and insurance coverage, and more specifically, how healthcare reform has impacted coverage. Long-standing disparities in coverage have consistently been a topic of discussion with multiple factors being considered as the cause for such differences. On one hand, studies have identified insurance coverage as an important determinant for disparities in access to care [2, 3]. While, lack of health care access and insurance coverage could be major contributors to poverty disparities, as this decreases one’s quality of life due to prolonged negative impacts of poverty and insurance status [4, 5]. Policies that reduce disparities in health insurance coverage are likely to have a broader effect on economic inequality [6]. Thus, it is imperative to examine the role of poverty and its association with access to care, to gain a deeper understanding of the disparities in the healthcare sector in order to improve health equity.

Implemented in 2014, evidence of the Affordable Care Act (ACA) showed a significant decrease in the uninsured rate from 18 to 12 % [7]. The expansion of health insurance, both public and private, resulted in a net increase of 16.9 million people gaining coverage between 2013 and 2015, allowing millions of previously uninsured individuals to access and utilize health care services [8]. The Medicaid expansion provision and the ACA market exchange subsidy contributed greatly to this sharp decline. As one of the major provisions of ACA, Medicaid expansion reduced health inequity by increasing access to health insurance coverage among low-income populations, who were at high risk of being uninsured. Medicaid expansion expanded the enrollment eligibility criteria for Medicaid to 138 % Federal Poverty Level (FPL), [9] resulting in gains in coverage for millions of low-income adults in more than 30 states [10]. After the debate on whether to expand Medicaid vs. weighing alternative approaches (i.e. using private insurance or increasing cost-sharing), more states have continued to expand Medicaid because data shows that low-income individuals who have historically experienced suboptimal access to care or gone without coverage, can benefit greatly from expansion [11–13].

Previous literature has examined the impact of Medicaid Expansion across various vulnerable populations (i.e. young mothers including pregnant women, veterans, people with disabilities, people with obesity, smokers, and immigrants) [14–20], disease conditions (i.e. cancers, AIDS and mental diseases) [21–30], socio-demographics [31, 32], and healthcare services (i.e. inpatient, outpatient and preventive services) [33–36], after the policy was implemented. Additionally, state-level analyses have been conducted to assess the impacts of expansion, as Kentucky and Indiana have been well discussed [37, 38].

Regarding the policy impact on insurance coverage rates and healthcare access, several researchers have examined this topic and concluded that the rate of persons insured increased due to Medicaid expansion in all cases. Huguet and colleagues examined the changes in uninsured visits across 412 primary care community health centers using electronic health records between 2012 and 2015 [39]. Blumberg et al. (2016) provided an overview of the characteristics of newly insured due to the ACA and those remaining uninsured in 2016 [40]. Other studies analyzed the insurance coverage gain of Medicaid expansion along with ACA by 2015 or 2016 [41–45]. Across each of these studies, researchers found that the rate of persons insured increased due to Medicaid expansion in all cases.

However, these studies only restricted the policy impact to the first- and second- year following Medicaid expansion in 2014 and did not distinguish Medicaid Expansion from other ACA provisions (i.e., market exchange subsidy, young adults remaining on parents’ health insurance plans until they reach age 26, etc.). The ACA market exchange subsidy eligibility is based on income, where individuals must earn at least 100 % FPL (above 138 % FPL in states that have expanded Medicaid), but no more than 400 % FPL. This premium subsidy can increase access to insurance for those between 100 and 400 % FPL. Therefore, it must be distinguished from the Medicaid expansion provision when analyzing the Medicaid expansion policy alone. Additionally, as different states adopted Medicaid expansion at different times, the dynamic effects seen across adoption of the policy have not been clearly examined in previous research.

To address these gaps, this study aimed to document changes in health insurance coverage for nonelderly US adults, aged 26–64, from 2012 to 2018 and evaluate the effects of the dynamic adoption of Medicaid expansion on poverty disparities in health insurance coverage at the national level. With a longer post-policy period, our study aimed to use causal inference to capture precise estimations of the sole effects of the Medicaid Expansion provision.

### Conceptual Model

**Fig. 1:**
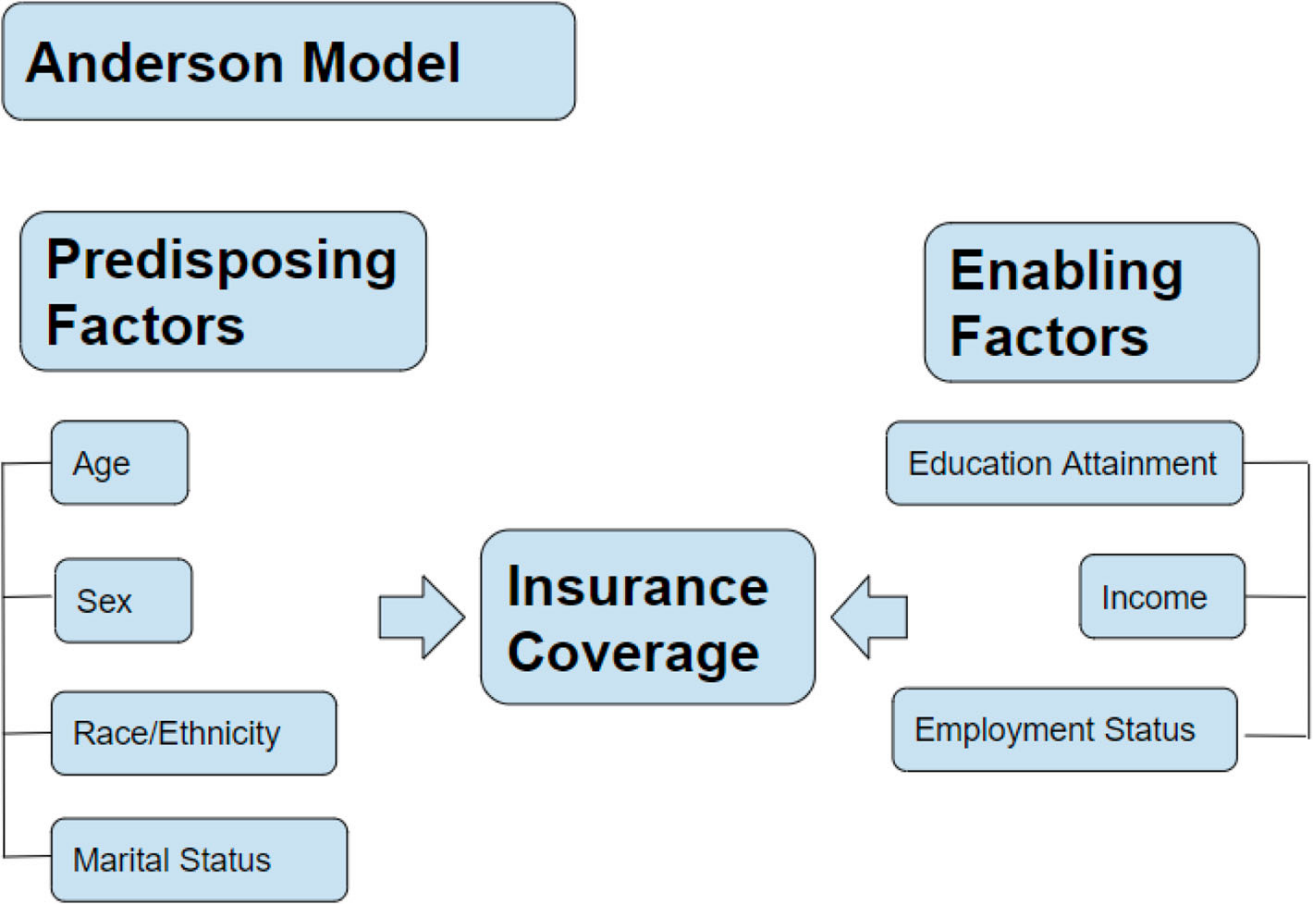
Conceptual Framework: Anderson Model

To address this study objective, the Andersen Behavioral Model for access to health care, derived from the original Anderson Healthcare Utilization Model, was adapted for this study [46]. This model illustrates that there are certain factors that increase one’s likelihood of using health care services and these are determined by three mechanisms of action: predisposing factors, enabling factors, and need. “Predisposing factors” are defined as demographic variables such as age, sex, race/ethnicity, marital status, education, employment, and poverty/income. The “enabling factor” in this study would be insurance coverage, and the “need” is access to health care services (Fig. 1).

## Methods

### Data

This study used publicly available data that was waived from review by the Institutional Review Board at Tulane University. Data analysis was based on repeated cross-sectional national population level data from the 2012–2018 7-year American Community Survey (ACS) Public Use Microdata Sample (PUMS) files. ACS is a very large survey, in terms of number of respondents who complete the survey, making it possible to obtain estimations for narrowly defined subpopulations by FPL [47]. PUMS are a sample of actual responses from the ACS and include most population and housing characteristics. This data source, with the flexibility to prepare customized tabulations, can be used for detailed research and analysis. In the PUMS, files have been de-identified to protect the confidentiality of all individuals and households. Further, this survey includes information about socio-economic, demographics, and other characteristics. The PUMS file contains over 3.5 million respondents each year with an average response rate of over 97 %. The differences in this cross-sectional survey were constant over time, which makes the estimation of trends in insurance coverage comparable [48].

### Analytic Strategy

This study employed differences-in-differences-in-differences (triple-D) design by comparing the outcome (health insurance coverage), before and after the intervention (implementation of Medicaid expansion) for the treatment group (individuals under 138 % FPL) and control group. This quasi-experimental design has been widely used to estimate the effects of a specific intervention or treatment by comparing the changes in outcomes over time between a population that is enrolled in a policy/program (i.e. the intervention group) and a population that is not (i.e. the control group) [49, 50]. The approach removes biases in post-policy period comparisons between the treatment and control group that could be the result from permanent differences between those groups, as well as biases from comparisons over time in the treatment group that could be the result of trends due to other causes of the outcome.

In this study, we did not include data prior to 2012 and excluded individuals less than 26 years old, as a provision of the ACA enables young adults to remain on their parents’ insurance plans until 26 years old. Without capturing effects of that provision, which have been well studied in previous research, our results excluded this potential confounding impact [51–53]. Additionally, a 0 to 1 intensity was used to demonstrate the effect of the dynamic enrollment of adoption of the policy for each state in order to generate more precise estimates.

The first regression was performed with the entire sample, with the treatment group being those under 138 % FPL and the control group being those with FPL above 138 %. The second regression included those under 138 % FPL as the treatment group and those above 400 % FPL as the control group. Those with an FPL range from 138 to 400 % were excluded in order to eliminate the ACA market exchange subsidy influence on the insurance coverage rate. The equation is as follows:

1 $$ \begin{array}{*{20}l} (1) &{Uninsured}_{isy}=\beta_{0} + \beta_{1}{Expanded}_{isy} + \beta_{2}\gamma_{sy} + \beta_{3}\delta_{is} + {\beta}_{4}\eta_{iy}+ \beta_{5}{Poverty}_{isy} + \beta_{6}{State}_{iy}+ \\ & \beta_{7}{Year}_{is} + {\beta_5X}_{isy} + \epsilon_{isy} \\ (2) & {Expanded}_{isy} = {Medicaid\_Expansion}_{sy}\ast{Poverty}_{isy}\end{array}$$

### Variables and Measures

*Uninsured*_*isy*_ was the study outcome. It was derived by the survey question “whether the individual has any insurance coverage” and was transformed into a dichotomous variable where 1 equaled no health insurance and 0 equaled having any insurance.

*Expanded*_*isy*_, variable of interest, was the interaction term that measured the individual level of Medicaid expansion status. This variable was calculated by *Medicaid_Expansion*_*sy*_ times *Poverty*_*isy*_. In the first regression, the coefficient of this variable reflected the impact Medicaid expansion had on insurance coverage between persons below 138 % FPL (treatment group) and above 138 % FPL (control group) in year $i$and state $s$. In the second regression, the control group were the individuals above 400 % FPL.

*Medicaid_Expansion*_*sy*_ was the Medicaid expansion policy indicator variable, defined as a varying intensity variable from 0 to 1. When a state expanded Medicaid, this variable equaled 1, otherwise, it equaled 0. For states that adopted the policy at the beginning of the year, time before this certain year was the pre-policy period and from this year to 2018 was the post-policy period. Coverage under Medicaid expansion became effective January 1, 2014 in all states that adopted the policy except the following: Michigan (4/1/2014), New Hampshire (8/15/2014), Pennsylvania (1/1/2015), Indiana (2/1/2015), Alaska (9/1/2015), Montana (1/1/2016), Louisiana (7/1/2016) [54]. For the above states, the indicator variable was coded as the portion of the year and then equaled to 1 in the following post-policy period. Specifically, the treatment for Michigan in 2014 was 275/365; the treatment for New Hampshire in 2014 was 138/365; the treatment for Indiana in 2015 was 334/365; the treatment for Alaska in 2015 was 122/365; and the treatment for Louisiana in 2016 was 184/365. Substantive Medicaid expansion policies prior to the state’s official implementation date of Medicaid expansion were not considered in the study. However, the prior adoption of Medicaid expansions in some states (IN, ME, TN, and WI) were quite limited with capped or closed enrollment. In some states a mild form of Medicaid expansion was adopted prior to the enactment of the ACA for both parents and childless adults (DE, DC, MA, NY, VT), which turned out to be an equivalent of the ACA expansion.

*Poverty*_*isy*_, was defined as a categorical variable using FPL threshold. FLP was calculated using poverty guidelines (one of the federal poverty measures) and the number of persons living in a household [55]. The poverty guidelines are updated each year by the US Department of Health and Human Services, for use for administrative purposes (e.g., determining financial eligibility for federal Medicaid programs). In the first regression, 138 % FPL was used as the cut-off point, where 1 was the population below 138 % FPL and 0 represented the population above 138 % FPL (control group). In the second regression, 1 represented those below 138 % FPL and 0 represented those above 400 % FPL (control group).

${\gamma }_{sy}$ was the interaction term for year and state.${\delta }_{is}$ was the interaction term for whether the individual was under 138 % FPL in a specific state. ${\eta }_{iy}$ was the interaction term for whether the individual was under 138 % FPL in a specific year (2012–2018).${State}_{iy}$ indicated the residence of state for the individuals in a given year. ${Year}_{is}$ represented the calendar year for the individuals in a state.

${X}_{isy}$was a series of individual demographic and socioeconomic covariates based on the Andersen model. Age, sex, race/ethnicity, marital status, education attainment, and employment status were adjusted in each model and these variables have been widely used in the literature to examine health care access and utilization [4, 5, 56, 57]. Age was measured as a categorical variable: 26–34, 35–44, 45–54 and 55–64. Sex was also a categorical variable, where 1 was male and 0 was female. Survey participants were asked to self-report their race (White, Black/African American, American Indian/Alaska Native, Asian, Native Hawaiian/other Pacific islander, or multiple race) and ethnicity (Hispanic/Latino: yes/no) separately. A 4-category race/ethnicity variable was coded as follows: participants reporting Hispanic/Latino ethnicity were considered Hispanic, regardless of race; all non-Hispanics were categorized as White, Black, or other races. Thus, Whites were coded as 0, Blacks, Hispanics, and Others were coded as 1, 2 and 3, respectively. Marital status was self-reported and categorized as married, widowed, divorced, and separated, and never married. For the model, we coded married as 0 and combined widowed, divorced, and separated into one group, and coded this group as (1) Self-identified education level was grouped by less than high school coded as 0, high school graduate, GED, or alternative coded as 1, and bachelor’s degree or higher coded as (2) Current employment status was categorized as unemployed coded as 0, employed coded as 1, and not in the labor force coded as 2.

Robustness checks were performed to test whether Medicaid expansion had an effect [58, 59]. To test this, we verified whether the control group (individuals with < 138 % FPL in non-expansion states) served as a good comparison for the treatment group (individuals with < 138 % FPL in states that expanded Medicaid). Therefore, we tested if the trends in the two groups were parallel before the policy was implemented. This assumption was tested indirectly by employing an event study model that interacted the treatment variables with the full set of fixed year effects. The regression took the form as Eq. 2 below. To satisfy the parallel trends assumption, no statistical significance on the first coefficient was expected, which suggested no change associated with Medicaid expansion between the period at least 2 years and 1 year prior to expansion. Statistical significance in later years’ coefficients indicated the expansion effected the treatment group with the given poverty level. In addition, we also examined the association of Medicaid expansion with a placebo outcome unrelated to insurance status that would not be affected by the policy. The placebo outcome was set as the probability of getting higher education. The placebo outcome check was performed in the same format as Eq. 1.

2 $$ {Uninsured}_{isy}={\beta }_{0}+{\beta }_{1}{Pre2*Poverty}_{isy}+{\beta }_{3}{Same0*Poverty}_{isy}+{\beta }_{3}{Post1*Poverty}_{isy}+{\beta }_{4}{Post2*Poverty}_{isy}+{\beta }_{5}{\gamma }_{sy}+{\beta }_{6}{\delta }_{is}{+ \beta }_{7}{\eta }_{iy}+{\beta }_{8}{Poverty}_{isy}+{\beta }_{9}{State}_{iy}+{\beta }_{10}{Year}_{is}+{\beta }_{11}X_{isy}+{\epsilon }_{isy} $$

Similar to the main estimating equation (Eq. 1), only the *Expanded*_*isy*_ treatment variable was changed from the main model into a set of time dummies for individuals given the year and state ($Pre2, Same0, Post1,Post2$, time ${Poverty}_{isy}$, respectively) in Eq. 2. The $Pre2$ term was an indicator variable set as 1 and represented at least 2 years before a state expanded Medicaid and 0 otherwise. The second $Same0$was 1 when in the year a state expanded Medicaid and 0 otherwise. Same algorithm followed for the $Post1$ and $Post2.$ The omitted category $Pre1$ was the year immediately before expansion. Thus, the coefficients on each of these variables gave the change for 2-year before expansion and 2-year post expansion. Other variables in Eq. 2 represented the same state/year fixed effects and control vectors as Eq. 1. Standard errors were clustered at the state level to account for state-level differences and serial autocorrelation. The 7-year data was a multi-year combination of the 1-year PUMS file with appropriate adjustments to the weights and inflation adjustment factors. The significance level for tests was set as 0.05. The data was weighted by the ACS survey weight and analyses were performed using SAS 9.4 and Stata 12.0.

## Results

**Fig. 2:**
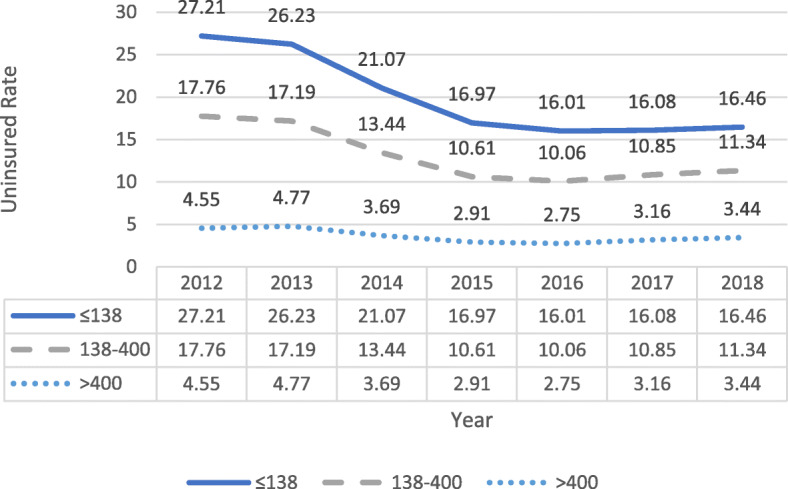
Uninsured rate trends from 2012 to 2018 by poverty level

Figure 2 presents the trends in the uninsured rate from 2012 to 2018 by poverty level groups. The uninsured rates reduced significantly after the market exchange subsidy and implementation of Medicaid expansion for all poverty levels. Specifically, compared with the uninsured rates in 2012, the uninsured rates in 2018 decreased by 10.75, 6.42, and 1.11 % points for individuals under 138 % FPL, between 138 and 400 % FPL, and above 400 % FPL, respectively. Although the uninsured rate decreased for all groups, those under 138 % FPL still had the highest uninsured rates.

Table 1 presents the pre-policy period comparison on demographic and socioeconomic characteristics between states who adopted Medicaid expansion on January 1, 2014 with states who did not adopt the policy at that time. Both adopted and non-adopted states presented similar age and gender distributions with more than 50 % people being over 45 years old and about 51 % female. Majority of the population were married and employed. White was the majority race/ethnicity while Black made up 8 % in adopted states and 14 % in non-adopted states. Overall, Medicaid expansion states had a higher education level (35 % received Bachelors’ degree or higher). Expanded states also had a much higher mean FPL than states without expansion (423 % FPL vs. 329 % FPL, respectively). Chi-square tests for categorical variables and two sample t-test for continuous variables were performed to explore the differences between adopted and non-adopted states by year 2014. All characteristics except gender showed statistically significant differences among adopted states and non-adopted states before Medicaid expansion took effect.

**Table 1:** Demographic Characteristics among adopted states and non-adopted states before 2014

		Adopted on 2014/1/1		Not adopted on 2014/1/1		*P*-value
		N	%	N	%	
age	26-34	293,965	19.93	285,258	19.58	<.0001
	35-44	332,162	22.52	330,374	22.68	
	45-54	423,514	28.71	417,549	28.66	
	55-65	425,556	28.85	423,645	29.08	
Gender	Female	754,787	51.17	745,841	51.2	0.5939
	Male	720,410	48.83	710,985	48.8	
Race/Ethnicity	White	1,061,213	71.94	1,088,222	74.7	<.0001
	Black	118,943	8.06	199,898	13.72	
	Hispanic	160,728	10.9	108,649	7.46	
	Other	134,313	9.1	60,057	4.12	
Marital Status	Married	896,448	60.77	903,190	62	<.0001
	Widowed/Divorced/Separated	257,902	17.48	285,617	19.61	
	Never married	320,847	21.75	268,019	18.4	
Education Level	Less than high school	124,888	8.47	140,152	9.62	<.0001
	High school graduate, GED, or alternative	837,969	56.8	887,294	60.91	
	Bachelor's degree or higher	512,340	34.73	429,380	29.47	
Employment Status	Unemployed	79,270	5.37	71,171	4.89	<.0001
	Employed	1,056,853	71.64	1,018,495	69.91	
	Not in labor force	339,074	22.98	367,160	25.2	
		Mean	Standard Error	Mean	Standard Error	
Federal Poverty Level		423.06	508.10	368.80	434.30	<.0001

Table 2 displays the results of the two regressions based on the triple-D design, each with a basic linear model and multivariate linear probability model with controlled covariates. For the first basic regression, those under 138 % FPL versus those above 138 % FPL, there was a 2.44 % point decrease in uninsured risk after expansion among those under 138 % FPL in adopted states versus control states. Controlling for socio-demographic characteristics, there was a decrease of 2.54 % points in uninsured risk which almost doubled the uncontrolled model’s reduction. For the second regression comparing those under 138 % FPL and above 400 % FPL, the unadjusted model showed a decrease of 3.09 % points in the uninsured probability while the adjusted model showed a decrease of 3.19 % points. Results also indicated individuals under 138 % FPL were still 12.78 % points and 19.77 % points more likely of being uninsured under expansion compared to those above 138 % FPL and above 400 % FPL, respectively. Further, as age increased, the likelihood of being insured also increased. Among all race and ethnicities, compared with whites, Hispanics were the most unlikely to have insurance.

**Table 2:** Unadjusted and adjusted triple-D estimates

		≤138% FPL VS >138%FPL						≤138% FPL VS >400%FPL					
Variable		Unadjusted			Adjusted			Unadjusted			Adjusted		
		Percentage Estimate	Standard Error	p-value	Percentage Estimate	Standard Error	p-value	Percentage Estimate	Standard Error	p-value	Percentage Estimate	Standard Error	*p*-value
Expanded		-2.44	0.0056	<.0001	-2.54	0.0057	<.0001	-3.09	0.0073	<.0001	-3.18	0.0072	<.0001
Poverty	>138% FPL	ref			ref								
	<=138% FPL	21.08	0.0021	<.0001	12.78	0.0057	<.0001						
	>=400% FPL							28.01	0.0023	<.0001	19.77	0.0055	<.0001
age	26-34				ref								
	35-44				-1.91	0.0012	<.0001				-1.63	0.0009	<.0001
	45-54				-3.58	0.0029	<.0001				-3.66	0.0030	<.0001
	55-65				-6.53	0.0052	<.0001				-6.7	0.0054	<.0001
Gender	Female				ref								
	Male				3.16	0.0012	<.0001				3.43	0.0014	<.0001
Race/Ethnicity	White				ref								
	Black				0.45	0.0020	<.0001				0.87	0.0021	<.0001
	Hispanic				3.57	0.0070	<.0001				3.04	0.0065	<.0001
	Other				2.34	0.0052	<.0001				1.96	0.0042	<.0001
Marital Status	Married				ref								
	Widowed/Divorced/Separated				5.22	0.0020	<.0001				3.13	0.0020	<.0001
	Never married				6.69	0.0029	<.0001				4.41	0.0030	<.0001
Education Level	Less than high school				ref								
	High school graduate, GED, or alternative				-7.52	0.0070	<.0001				-6.58	0.0073	<.0001
	Bachelor's degree or higher				-14.24	0.0124	<.0001				-13.07	0.1257	<.0001
Employment Status	Unemployed				ref								
	Employed				-20.36	0.0124	<.0001				-12.52	0.0122	<.0001
	Not in labor force				-16.4	0.0095	<.0001				-15.05	0.0090	<.0001
Federal Poverty Level					-0.002	0.0000	<.0001				0	0.0000	<.0001

**Fig. 3:**
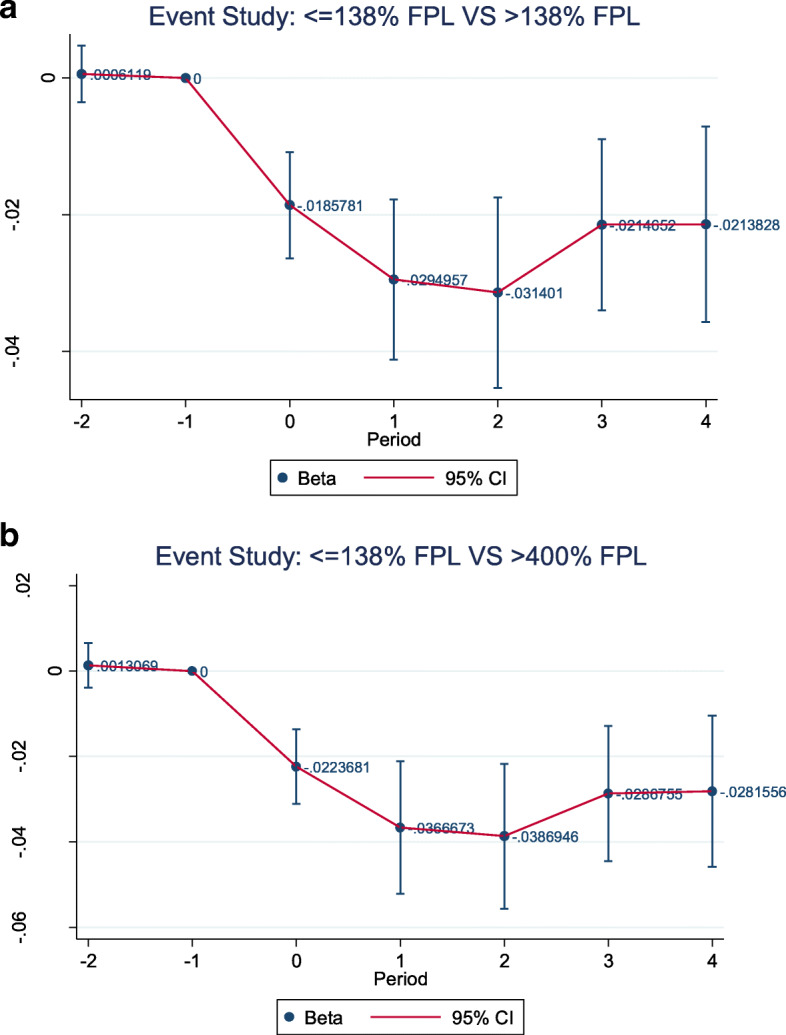
**a** Event Study - ≤138 % FPL VS > 138 % FPL. **b** Event Study - ≤138 % FPL VS > 400 % FPL

Figure 3a and b present event study graphs that meet the parallel trends assumption in the pre-policy period between the adopted states and the control states. The graphs also showed that if Medicaid expansion had not occurred, changes in insurance coverage would have not been correlated with pre-treatment uninsured rates. The placebo outcome robustness check reported insignificant results with *p*-values equal to 0.634 for regression ≤ 138 % FPL and > 138 % FPL, and 0.545 for regression ≤ 138 % FPL and > 400 % FPL, respectively. With both regressions’ p-values greater than 0.05, placebo checks showed that Medicaid expansion did not have an impact on our outcome, unrelated to the policy.

## Discussion

After a long post-Medicaid expansion period, our study examined the effects of the dynamic adoption of Medicaid expansion on poverty disparities in health insurance coverage at the national level. Findings from this study demonstrate that trends in the uninsured rate for each FPL group decreased from 2012 to 2018. Across all 7 years, the “over 400 % FPL” group continuously had the lowest uninsured rate while the “under 138 % FPL” group had the highest uninsured rate. For all FPL groups, the uninsured rates were reduced significantly after implementation of Medicaid expansion. Through the triple-D design, both regressions showed a significant reduction in uninsured risk was due to Medicaid expansion.

This study documented that a large increase in Medicaid eligibility was associated with a significant decrease in the uninsured rate during the study period. We also found that the population with the largest coverage gains from expansion were those aged 55–64 years old, well-educated, and employed. For example, our results found that individuals with bachelor’s degree or higher were 14.24 % less likely to be uninsured compared with individuals who had less than a high school education. These findings may influence states’ decisions with respect to Medicaid expansion.

Previous studies have identified the benefits of Medicaid expansion on health insurance coverage among individuals with low socioeconomic status. However, this study further added to the literature with novel contributions. The overall results of this study extended the literature in several ways. First, this paper presented new evidence used a large national level survey and longer time frame to assess the association of expansion and insurance coverage. Many datasets such as the Behavioral Risk Factor Surveillance System, Gallup-Healthways Well-Being Index (daily national telephone survey), and federal survey data, have been used to assess the socioeconomic disparities in health care access [43–45, 56]. Studies using these datasets found that health care access for people in lower socioeconomic strata had higher improvement in states that expanded eligibility for Medicaid under the ACA than states that did not. Socioeconomic disparities in health care access narrowed significantly under the ACA. Rather than assessing the early stage of the first- and second- year of Medicaid expansion, our study reached similar conclusions using comparable study design but in unrelated, population-based survey data. In addition, to report the association between Medicaid expansion and insurance coverage among low-income populations, our study demonstrated a more precise estimation of this relationship by using a varying intensity policy variable and eliminating the effect of parents’ health insurance coverage for young adults under 26 years of age and the ACA premium subsidy. Sommers et al. found that increasing insurance coverage increased health care utilizations by conducting a triple-D analysis of survey data from November 2013 through December 2015 on US citizens ages 19 to 64 years old with incomes below 138 % FPL in Kentucky, Arkansas, and Texas [45]. Our findings were consistent with this state-level research and strengthened the conclusion at a national level that poverty disparities were narrowed by increases in insurance coverage.

Secondly, the triple-D estimates started with the average time changes for the population under 138 %FPL in the expanded states, then netted out the change in means for the population under 138 %FPL in the control states and the change in means for the population over 138 %FPL in the expanded states. This controlled for two types of potentially confounding trends: changes in insurance coverage for the population under 138 %FPL across states (that would have nothing to do with the policy) and changes in health status of all people living in the expanded states (possibly due to other state policies that affect everyone’s insurance status, or state-specific changes in the economy that affect everyone’s insured rate). The triple-D design allowed us to estimate the casual impact of Medicaid expansion and contributed to an evidence-based policy decision-making process.

This study is not without its limitations. First, due to data availability, we were only able to estimate the effects up until 2018. Therefore, some states, such as Louisiana, had a very short post-policy period. Additionally, for states that adopted Medicaid expansion after 2018, their effects were not seen. As future waves of data become available, it is worthwhile to revisit these estimates. Another limitation of this study is that detailed policy differences within each state were not considered. Some states had different eligibility criteria for Medicaid prior to the adoption of expansion. In other words, the policy impact varied in the adopted states due to the before-adoption eligibility criteria. Further studies may examine these differences to get the unbiased policy effect. Lastly, ACS did not contain detailed health information such as disease conditions and healthcare utilization. Controlling for pre-conditions of individuals, could improve the precision of our estimates, and with specific healthcare utilization measurements, we could better understand the direct impact of this policy on healthcare utilization.

## Conclusions

In this study, health insurance coverage improved substantially among populations with income less than 138 % FPL. Overall, Medicaid expansion has made impressive strides in reducing health inequity with increasing access to health insurance coverage among low-income populations. Our findings are consistent with previous literature and reflect the achievement of Medicaid expansion’s goal, making healthcare accessible for low-income populations. However, individuals under 138 % FPL are still more likely to be uninsured.

Unlike other developed countries, the US does not have universal health insurance programs in which the government plays a dominant role. One major challenge of the US healthcare system is providing equitable access to care. Disparities in health outcomes by income status are persistent and hard to reduce in the US. Therefore, the ACA and Medicaid expansion are critical to expand health insurance to improve overall quality of life for US citizens. Based on our findings, adequate access to health care services still falls short and there is still a long way to go to achieve healthcare equity. Healthcare professionals and policy makers should continue to advocate for increased coverage for low-income populations. Reducing health inequity in health insurance coverage is achievable but will require better outreach to the remaining uninsured, particularly among vulnerable groups with historically low and disproportionate uninsured rates.

## Data Availability

The American Community Survey data is public data available on the ACS official website.

## Abbreviations

ACA: Affordable Care Act
ACS: American Community Survey
FPL: Federal Poverty Level
PUMS: Public Use Microdata Sample files
Triple-D design: Differences-in-differences-in-differences design
DC: District of Columbia
DE: Delaware
IN: Indiana
MA: Massachusetts
ME: Maine
NY: New York
TN: Tennessee
VT: Vermont
WI: Wisconsin
